# A Novel Frameshift Variant and a Partial *EHMT1* Microdeletion in Kleefstra Syndrome 1 Patients Resulting in Variable Phenotypic Severity and Literature Review

**DOI:** 10.3390/genes16050521

**Published:** 2025-04-29

**Authors:** Maria Tzetis, Anastasios Mitrakos, Ioanna Papathanasiou, Vasiliki Koute, Konstantina Kosma, Roser Pons, Aspasia Michoula, Ioanna Grivea, Aspasia Tsezou

**Affiliations:** 1 Laboratory of Medical Genetics, Medical School, National and Kapodistrian University of Athens, Thivon & Levadias, 11527 Athens, Greece; mtzetis@med.uoa.gr (M.T.); amitrakos@med.uoa.gr (A.M.); kokosma@med.uoa.gr (K.K.); 2 Medical Genetics Laboratory, GeneTech Analytics Ltd., 41 Asklepiou, 41222 Larissa, Greece; 3 University Research Institute for the Study of Genetic and Malignant Disorders in Childhood, National and Kapodistrian University of Athens, Thivon and Levadias, 11527 Athens, Greece; 4 Laboratory of Cytogenetics and Molecular Genetics, Faculty of Medicine, University of Thessaly, 3 Panepistimiou, Biopolis, 41500 Larissa, Greece; iopapat@uth.gr; 5 Pediatric Neurology Outpatient Clinic, Department of Pediatrics, University Hospital of Larissa, 41500 Larissa, Greece; koutevas@yahoo.gr; 6 First Department of Pediatrics, Medical School, “Aghia Sophia” Children’s Hospital, National and Kapodistrian University of Athens, Thivon and Papadiamantopoulou, 11527 Athens, Greece; roserpons@med.uoa.gr; 7 Department of Pediatrics, Faculty of Medicine, University of Thessaly, Biopolis, 41500 Larissa, Greece; amichoula@gmail.com (A.M.); iogrivea@uth.gr (I.G.)

**Keywords:** array comparative genomic hybridization (aCGH), microdeletion 9q34.4, Kleefstra 1 syndrome (KLEFS1), exome sequencing (ES)

## Abstract

Background: Kleefstra syndrome 1(KLEFS1, OMIM#610253) is a rare neurodevelopmental disorder (NDD) instigated by heterozygous variants or microdeletions occurring in the 9q34.4 genomic region of the euchromatic histone methyltransferase-1 (*EHMT1*) gene and is inherited in an autosomal dominant (AD) manner. The clinical phenotype of KLEFS1 includes moderate to severe intellectual disability (ID), hypotonia, and distinctive facial features and additionally involves other organ systems (heart, renal, genitourinary, sensory) albeit with phenotypic heterogeneity between patients. The purpose of this study is to expand the genotypic spectrum of KLEFS1 and compare phenotypic features of the syndrome of already published cases. Methods: Exome sequencing (ES), chromosomal microarray analysis (CMA), as well as sanger sequencing, for confirmation of the de novo status of the frameshift variant, were used. Results: Here we describe two more cases, both males with a similar age and carriers of novel variants; one with a frameshift variant involving exon 13: p.Val692Glyfs*64 and the other with the smallest so far described, 11 Kb (exons 19-25), 9q34.4 microdeletion: 9q34.3 (140703393-140714454). Both presented with an NDD disorder with one showing more severe ID with significant social disabilities, while the other with the microdeletion had mild ID and following a normal education curriculum. Neither of them were obese nor had any other significant organ system disorder. Conclusions: The observed phenotypic variability due to genotypic differences in the two children contributes to the expanding spectrum of KLEFS1 disease phenotypes.

## 1. Introduction

Kleefstra syndrome (KLEFS1, OMIM #610253) is a rare, autosomal dominant neurodevelopmental disorder with a recently re-calculated prevalence of 1:36,000 [1]. The syndrome is characterized by intellectual disability (ID), global developmental delay (GDD), hypotonia, attention deficit hyperactivity disorder (ADHD), and distinctive facial features, with epilepsy/seizures, cardiac defects, genital anomalies, hearing loss, and behavioral and sleep disorders being present as less common clinical characteristics, all documented in published case studies and reviews [1,2,3,4,5,6,7,8,9,10,11,12]. Additionally, KLEFS1 probands are at risk of developing severe psychiatric disorders, as well as regression of previous motor, language, and communication skills in adolescence (on average >18 yrs), with early sleep disturbances being a precursor for severe regression [13,14].

The majority of KLEFS1 cases are sporadic due to de novo heterozygous mutations (frameshift, nonsense, splice-site mutations) in the Euchromatin Histone Methyl Transferase 1 (*EHMT1)* gene, or microdeletions involving the 9q34.3 region (OMIM #607001). It has been reported that cases with microdeletions, partially or wholly deleting only the *EHMT1* gene, generally exhibit comparable phenotypic characteristics to those with intragenic variants [3,5,9,15,16]. The *EHMT1* gene includes 27 exons and encodes a histone methyltransferase, a pivotal component of the epigenetic machinery, that methylates lysine 9 on histone H3 (H3K9) with diverse domain specific functions. Loss of *EHMT1* function leads to reduced levels of H3K9 demethylation (H3K9me2), resulting in impaired synaptic function and neurodevelopment dysregulation of gene expression, especially those crucial for neuronal connectivity and function. Such disruptions affect cognitive function, behavior, and physical development [4,10,17].

Rare cases may involve pathogenic variants in other genes that interact with *EHMT1* or disrupt the same pathway, for example, KLEFS2 (OMIM#61768), caused by variants in the lysine-specific methyltransferase 2C gene (*KMT2C*, 606833) resulting in a phenotype overlapping with that of KLEFS1 [4,18].

Here we report on two additional patients of Greek origin, both with novel and de novo variants, a frameshift mutation in exon 13 of *EHTM1* gene and a partial *EHMT1* gene deletion involving exons 19-25 (11 Kb deletion).

## 2. Methods

### 2.1. Editorial Policies and Ethical Considerations

Informed consent was obtained from both patients’ legal guardians and the study was approved by the National and Kapodistrian University of Athens Bioethics committee.

### 2.2. DNA Extraction

DNA from the probands as well as parental DNA was extracted from peripheral blood lymphocytes in EDTA using the QiaSymphony SP robotic system and the QIAsymphony DSP Mini kit. Purity and concentration of the extracted DNA was determined using the NanoDrop 1000 UV-Vis spectrophotometer and Qubit 3 fluorometer (Thermo Fisher Scientific, Waltham, MA, USA).

### 2.3. Exome Sequencing (ES)

Exome sequencing for P1 was performed on an MGI DNBSEQ-T7 sequencer with the capture-based IDT xGen Exome Research v2 library preparation kit (Integrated DNA Technologies, Coralville, IA, USA), covering the coding regions of ~19.000 genes (~34 Mbp). A total of 9.118.152.750 bases were sequenced from a total of 60.787.685 reads (99.71% mapped reads). A total of 145,365 exonic variants were identified. In total, 97% of all coding regions had at least 20× coverage, while the mean coverage was at 99×. Bioinformatic analysis including alignment against GRCh37/hg19 version of the human genome and variant calling was performed with the “SEQ Platform (v8.10.0—https://seq.genomize.com/ Genomize, Ankara, Turkiye). Variant annotation was performed with the use of available databases, scientific literature, and in silico variant effect prediction tools including 1000 Genomes, gnomAD (ExAC), UMD-predictor, Shift, PolyPhen, Mutation Taster, ClinVar, VarSome, OMIM, and HPO.

### 2.4. Chromosomal Microarray Analysis (CMA)

Chromosomal microarray analysis was performed in P2 and his parents, with the high-resolution 4 × 180 K G3 CGH + SNP microarray platform (G5890A, Design ID 029830, Agilent Technologies, Santa Clara, CA, USA). The wet lab protocol was carried out following the manufacturer’s instructions and included enzymatic digestion of the genomic DNA sample as well as a sex-matched reference DNA, differential labeling with Cy5 fluorescent dye for the samples and Cy3 for the reference. After combining, the labeled DNA samples were applied to the microarray, hybridized for 24 h at 67 °C, washed, and scanned at 3 μm resolution (SureScan Dx, Agilent Technologies, Santa Clara, CA, USA). The .tiff images were extracted with Agilent Feature Extraction software v.12.2 and downstream analysis was performed with CytoGenomics v.5.0.2 software suite, utilizing the ADM-1 aberration detection algorithm. Evaluation and classification of Copy Number Variants (CNVs) was based on published literature as well as available databases were used, including Franklin Genoox (https://franklin.genoox.com, accessed on 1 April 2025), the Database of Genomic Variants (DGV), DECIPHER, and ClinVar. All variants were classified according to American College of Medical Genetics and Genomics (ACMG) and ClinGen guidelines [19].

### 2.5. Sanger Sequencing

Parental extracted DNA samples from P1 were subjected to targeted mutation sanger sequencing to determine the origin of the mutation. For the design of PCR primers the following databases were used: NCBI: https://www.ncbi.nlm.nih.gov/ (accessed on 1 April 2025), ENSEMBL: http://www.ensembl.org (accessed on 1 April 2025). The primers were designed using Primer 3 [20]. The primer sequences were as follows: *EHMT1* forward: 5′-CCGATTTCCTCCCCTGAAGT-3′, *EHMT1* reverse: 5′-TACTTTTCCGAGTCGAGGGC-3′ (NM_024757.5; product size 196 bp). The PCR protocol used for the amplification of the specific fragments was according to the manufacturer’s instructions (HotStarTaq MasterKit, Qiagen, Hilden, Germany). A single-step enzymatic clean-up of PCR products was performed (ExoSAP-IT kit, Affymetrix, High Wycombe, UK), followed by sequencing reaction of all PCR products in an ABI3500 sequencer (Applied Biosystems, Inc., New York, NY, USA) using the BigDye^®^ Terminator v3.1/Sequencing Standard Kit (Applied Biosystems, Inc., New York, NY, USA). The sequencing reaction was performed in both forward and reverse directions.

## 3. Case Reports

### 3.1. Patient 1 (P1)

P1 is a 7-year old male (DOB 15/12/2017) born to non-consanguineous parents. He was delivered prematurely at 35 weeks of gestation by caesarian section, due to an initially twin pregnancy complicated by the miscarriage of the 2nd embryo at the end of the first trimester and further complicated by placental abruption. His weight at birth was 2440 g (50th percentile) and his Apgar score was 8 at 1′ and 9 at 5′. His neonatal period was notable for a prematurity-related Grade I intraventricular hemorrhage, a 3 mm ventricular septum defect detected on echocardiogram that subsequently resolved completely, and dysmorphic features along with a small penis. He underwent an endocrine workup, molecular analysis of the androgen receptor gene, and conventional karyotype analysis, all of which were normal. His psychomotor development was delayed. He gained head control at 6 months, was able to sit at 12 months, and to walk independently at 2 years old. He spoke his first words at 2 years and used simple sentences by 4 years. A brain MRI at 1 year of age revealed enlarged frontal subarachnoid spaces. At the age of 6, he experienced focal seizures. His EEG showed spike-wave activity in the left temporal-occipital region with generalization. He was started on oxcarbazepine (15 mg/kg) as an antiseizure therapy, with a good response to date.

At 7 years old, he underwent evaluation at another institution and was diagnosed with moderate ID, ADHD, axial hypotonia, sleep disturbances, and frequent temper tantrums. Dysmorphic features observed on exam included hypertelorism, a broad nasal bridge, thick and broad nasal tip, short philtrum, a broad protruding forehead, and a small penis. His weight was at the 15th percentile, height at the 3rd percentile, and head circumference at the 3rd percentile for his age and gender. Εxome sequencing (ES) was performed and a pathogenic novel and de novo frameshift mutation was detected in exon 13 of *EHMT1* gene (NM_024757.5), c.2075-2097del (p.Val692Glyfs*64).

### 3.2. Patient 2 (P2)

P2 is a 9-year-old male, born to consanguineous parents who are second cousins. Delivery was at 38 weeks of gestation, his birth weight was 2915 g (25th percentile), length 48.5 cm (35th percentile), and head circumference 34 cm (50th percentile). His Apgar scores were 9 at 1 min and 10 at 5 min. His neonatal period was remarkable for an episode of apnea. His head ultrasound was normal.

His psychomotor development was delayed. He gained head control at 6 months, sat at 10 months, and walked independently at 22 months. He spoke his first words at 3 years of age. He experienced multiple episodes of otitis media, which required tympanostomy tube placement. Subsequent hearing testing was normal. Brain MRI showed increased perivascular spaces and a mega cisterna magna. Molecular genetic testing for fragile X syndrome (FRAX) and conventional karyotype analysis were normal.

At 5 years old, he was referred for neurological assessment. He was diagnosed with mild ID, ADHD, axial hypotonia, and dysmorphic features, including hypertelorism, a broad nasal bridge, tented upper lip vermillion, prominent nasal columella, pointed chin, and broad protruding forehead. His weight was at the 15th percentile, height at the 3rd percentile, and head circumference at the 3rd percentile for his age and gender. He underwent chromosomal microarray analysis (CMA), which revealed a pathogenic novel and de novo 11 Kb partial EHMT1 deletion spanning exons 19-25 of the gene, arr[GRCh37] 9q34.3(140703393-140714454)x1.

## 4. Results

While both patients were affected with KLEFS1, there was clinical heterogeneity between P1, SNV carrier, and P2 with the 9q34.3 microdeletion. Developmental and phenotypic features of these two patients are given above and are compared with KLEFS1 individuals reported in the literature with *EHMT1* pathogenic SNVs or 9q34 microdeletions in Supplementary Table S1.

**Figure 1:**
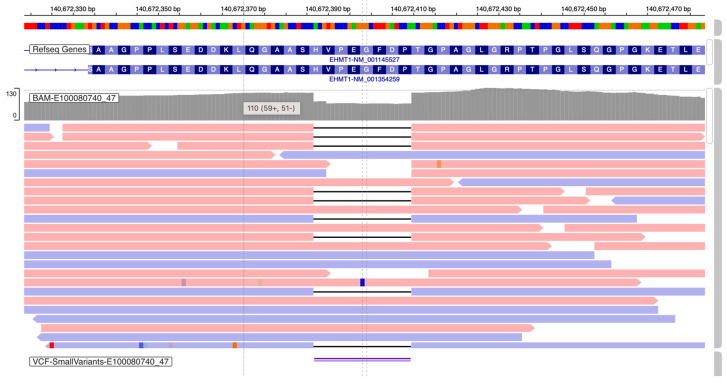
*EHMT1:* c.2075-2097del (p.Val692Glyfs*64), identified in P1 by ES (IGV image).

For P1, ES identified a novel heterozygous frameshift variant in exon 13 of *EHMT1* gene (NM_024757.5), c.2075-2097del (p.Val692Glyfs*64) (Figure 1). The truncating variant deletes the C-terminal ANK repeat and SET domain of the EHMT1 protein [1]. 

Sanger sequencing of the probands parents confirmed the de novo status of the variant (Figure S1). variant was classified as pathogenic based on the ACMG criteria (PVS1, PM2) [19]. No other causative pathogenic or likely pathogenic SNVs were detected in other loci, as investigated by ES.

**Figure 2:**
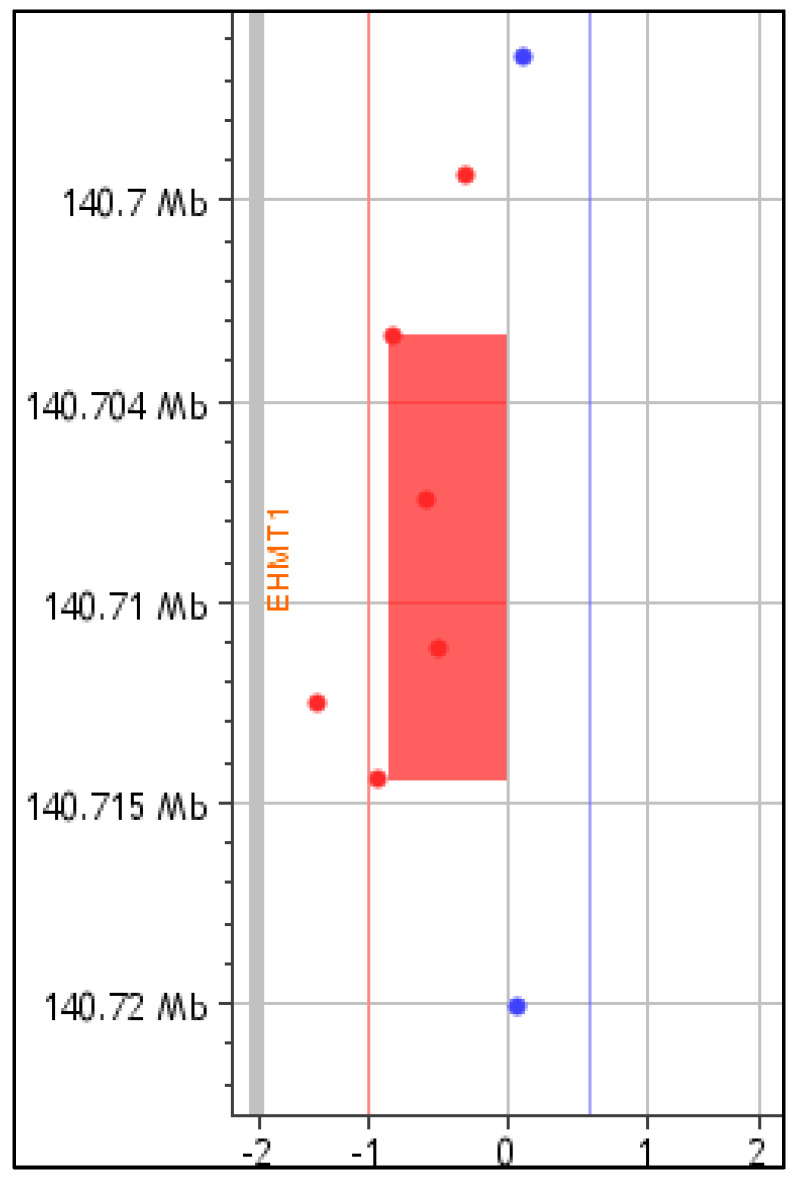
*EHMT1* microdeletion in P2 identified by CMA, spanning exons 19-25 (arr[GRCh37] 9q34.3(140703393-140714454)x1; 11Kb).

For P2, CMA identified a novel 11 Kb partial *EHMT1* deletion spanning exons 19-25 of the gene, arr[GRCh37] 9q34.3(140703393-140714454)x1, [(NC_000009.11:g.(?_140703393)_(140714454_?)del] (Figure 2). The microdeletion deletes the SET domain of the EHMT1 protein [1]. CMA testing of parental DNA confirmed the de novo status of the 9q34.3 microdeletion. This newly defined microdeletion was classified as pathogenic based on the ACMG and ClinGen criteria 1A, 2A-2E, 2H, 3C, and 4L. No other causative pathogenic or likely pathogenic CNVs were detected.

## 5. Discussion

Kleefstra syndrome (KLEFS1) belongs in the category of Mendelian disorders affecting the epigenetic machinery, as it results from pathogenic variants in components of the epigenetic apparatus. The *EHMT1* gene consists of a (RING)-like domain with unknown until now function (exons 11-12), the “reader” ankyrin repeat (ANKR) domain (exons 15-20), which is important for H3K9 methylation by the EHMT1/EHMT2 complex, the PreSET domain (N-terminal to SET domain) (exons 21-23), and the catalytic Su(var)3–9 Enhancer-of-zetste, trithorax (SET) domain (exons 24-26), involved in heterodimer formation with EHMT2 [1,11,21]. Recent studies have considerably advanced our understanding of the molecular mechanisms underlying KLEFS1. Recent studies in human iPSC-derived neuronal cells from KLEFS1 patients revealed that EHMT1 activity was necessary for the maintenance of different subcellular structures, as the Golgi apparatus, for functioning of the lysosomal system and the centrosome, opening new possibilities in understanding the functional impact of *EHMT1* haploinsufficiency in KLEFS1 patients. Additionally, a mechanism was revealed linking EHMT1 activity to the neuronal regulator NRSF/REST through an miRNA-dependent pathway and to enhanced NMDA receptor signaling, leading to aberrant neuronal gene regulation and altered neurodevelopment in human Kleefstra syndrome-patient iPSC-derived neuronal cells [17,22,23].

In the present manuscript we have analyzed the clinical features of two KLEFS1 patients of Greek origin, with variability in the severity of their phenotypic features. Patient 1 carries a novel frameshift variant in exon 13 [c.2075-2097del (p.Val692Glyfs*64)], resulting in a termination codon after 64aa and ultimately a truncated protein missing the C-terminal ANKR and SET domains, and therefore in *EHMT1* haploinsufficiency, a well-known KLEFS1 disease mechanism [11]. Patient 2 carries a novel 11 Kb partial *EHMT1* microdeletion, spanning exons 19-25, [(NC_000009.11:g.(?_140703393)_(140714454_?)del] eliminating the C-terminal SET domain of the gene. To the best of our knowledge, this specific deletion is the smallest identified by CMA until now and if it escapes nonsense-mediated decay (NMD), it would result in a loss of a significant part of the SET domain, likely leading to partial functional protein. The variant effect on the produced protein could potentially explain the milder phenotype of P2.

Since the identification of the *EHMT1* gene as mainly responsible for KLEFS1 [12], pathogenic SNVs or 9q34.3 microdeletions are increasingly being detected. Pathogenic variants are scattered throughout the gene, although clustering is observed within ANKR, as well as SET domains. It has been reported [1] that loss of the “reader” EHMT1 activity due to an ANKR disruptive protein altering variants is sufficient for the presence of KLEFS1 phenotypic features, similarly to *EHMT1* haploinsufficiency, while disruption of only the ‘‘writer’’ methyltransferase activity of the SET domain does not result in a typical KLEFS1 phenotype. In agreement with the above reports, P1 carrying the c.2075-2097del *EHMT1* frameshift variant resulting in lack of the C-terminal ANKR and SET domains, and likely resulting in haploinsufficiency, exhibits a more typical KLEFS1 phenotype. On the contrary, P2 carrying the partial *EHMT1* microdeletion presents a mild phenotype, following a mainstream school curriculum with additional special tutoring, although lagging behind his peers especially in terms of language development.

The role of the *EHMT1* gene in chromatin remodeling and gene expression regulation is crucial for normal neurodevelopment. Pathogenic variants leading to *EHMT1* haploinsufficiency or production of non-functional protein disrupt these processes resulting in the specific developmental and behavioral features seen in all published cases with KLEFS1 [1,2,6,14,15]. In addition to neurodevelopmental features, which seem to regress in adolescent patients with KLEFS1, as mentioned above, additional health issues, such as epilepsy with a prevalence of about 15%, are mentioned in large cohorts of KLEFS1 patients [4]. Congenital heart defects and early-onset arrhythmias have been recently observed in a significant number of individuals with KLEFS1 requiring close heart monitoring incorporated into routine follow-up care [24]. Additionally, a significant portion of KLEFS1 individuals exhibit hearing and gastrointestinal (GI) manifestations with variable severity [2,3,4,10,25]. Both of our patients (P1, 7 years old and P2, 9 years old) have neurodevelopmental and facial features and P1 additionally has epilepsy, heart defects, and a micropenis. Neither one has other systemic manifestations, such as ophthalmological issues, hearing loss, GI manifestations, renal manifestations, or musculoskeletal complications. Our patients are still too young to evaluate any psychiatric or motor regression issues.

KLEFS1 represents a very rare entity with only about 160 patients reported to date. The phenotypic variability observed in the two children with KLEFS1 presented above, along with the report of the smallest *EHMT1* microdeletion seen until now, contributes to the expanding spectrum in the disease phenotype of KLEFS1. Ongoing research efforts and additional reports of genotype-phenotype correlations in patients are essential to elucidate the underlying mechanisms of the syndrome and improve diagnostic accuracy.

## Data Availability

The original contributions presented in this study are included in the article/Supplementary Materials. Further inquiries can be directed to the corresponding author.

## Supplementary Materials

The following supporting information can be downloaded at: https://www.mdpi.com/article/10.3390/genes16050521/s1, Figure S1: Sanger sequencing of P1 and parental samples for detection of EHMT1 exon 13 variant (arrow depicts the start of the frameshift; P1: proband 1, M: mother, F: father). Table S1: Clinical characteristics of patients and comparison with those from the bibliography.
