# Supplementary material for: A Novel Frameshift Variant and a Partial EHMT1 Microdeletion in Kleefstra Syndrome 1 Patients Resulting in Variable Phenotypic Severity and Literature Review
Source: Genes (Basel). 2025 Apr 29;16(5):521. doi: 10.3390/genes16050521 (PMC12110755; doi:10.3390/genes16050521)
Supplement: Supplementary file 1 [file genes-16-00521-s001.zip › Fig S1(supplementary Fig 1).pdf]

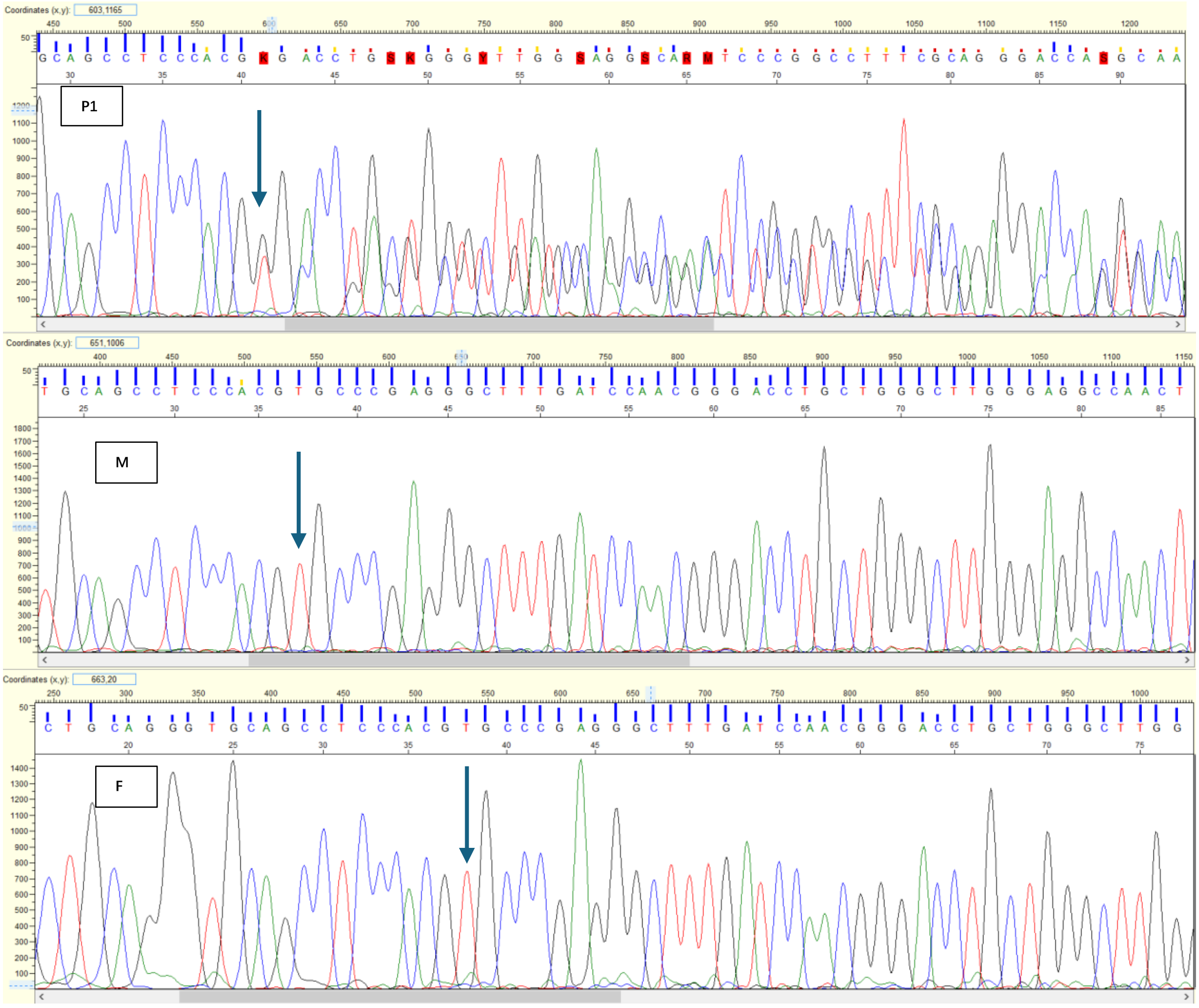

Figure S1: Sanger sequencing of P1 and parental samples for detection of *EHMT1* exon 13 variant (arrow depicts the start of the frameshift; P1: proband 1, M: mother, F: father)
