# Supplementary material for: A Novel Frameshift Variant and a Partial EHMT1 Microdeletion in Kleefstra Syndrome 1 Patients Resulting in Variable Phenotypic Severity and Literature Review
Source: Genes (Basel). 2025 Apr 29;16(5):521. doi: 10.3390/genes16050521 (PMC12110755; doi:10.3390/genes16050521)
Supplement: Supplementary file 1 [file genes-16-00521-s001.zip › genes-3608564-supplementary.pdf]

Table S1: Clinical characteristics of patients and comparison with those from the bibliography.

KLEFS1 clinical characteristics (# 610253)	**Patient 1** c.2075-2097del (p.Val692Glyfs*64) (DOB:15/12/2017)	**Patient 2** arr[GRCh37] 9q34.3 9140703393-140714454)x1/ (exons 19-25) (DOB:13/11/2015)	Frequency (%) of clinical characteristics from bibliography †
**GROWTH** Weight -Obesity	-	-	17-45%
**HEAD & NECK**			
Brachycephaly	-	-	40%
Microcephaly	+	+	13-38%
Flat face	+	+	55-80%
Prognathism	-	-	~50%
Coarse facies	+	+	25-50%
Malformed ears	-	-	50-80%
Hearing loss	-	-	15-50%
Hypertelorism	+	+	55-70%
Synophrys	-	-	55-80%
Anteverted nares	-	-	25-80%
Tented and cupid-bowed upper lip	+	+	25-75%
Everted lower lip	+	+	25-50%
Macroglossia	-	-	40-60%
**CARDIOVASCULAR** Heart/conotruncal heart defects	+	-	13-47%
**NEUROLOGIC** *Central Nervous System/Behavioral Psychiatric Manifestations*			
Intellectual disability	Severe	mild	50-100%
Hypotonia (HP:0001252)	++	+	13-86%
Seizures/ Epilepsy	+	-	13-38%
Behavioral problems	++	-	65-70%
Sleep disorder	++	-	38-53%
Stereotypic movements	-	-	0-18%
Obsessive-compulsive disorder/ ADHD	++	-	30-75%
Aggressive behavior	+	-	9-41%
**Miscellaneous**			

Abnormality of the skin	+	-	10-15%
Recurrent infections	-	+	12-57%
Congenital Kidney/ urinal abnormalities	-	-	15-30%
Genital anomalies in males	+ (micropenis)	-	30-40%

ADHD : Autism Deficit Hyperactivity Disorder, [†] Frazier et al., 2025; Kleefstra et al., 2012; Rots et al., 2024 ; Huang et al., 2021 ; Ciaccio et al., 2018 ; Willemsen et al., 2011.
